# Supplementary material for: Splice-Junction-Based Mapping of Alternative Isoforms in the Human Proteome
Source: Cell Rep. Author manuscript; Available in PMC 2020 Jan 15. (PMC6961840; doi:10.1016/j.celrep.2019.11.026)
Supplement: 2 [file NIHMS1546469-supplement-2.zip › DF1/PXD009737/TestisGluC/TestisGluC_17_DENND4B_ISLANPVDYFVVAGLAGNGAPIPE.pdf]

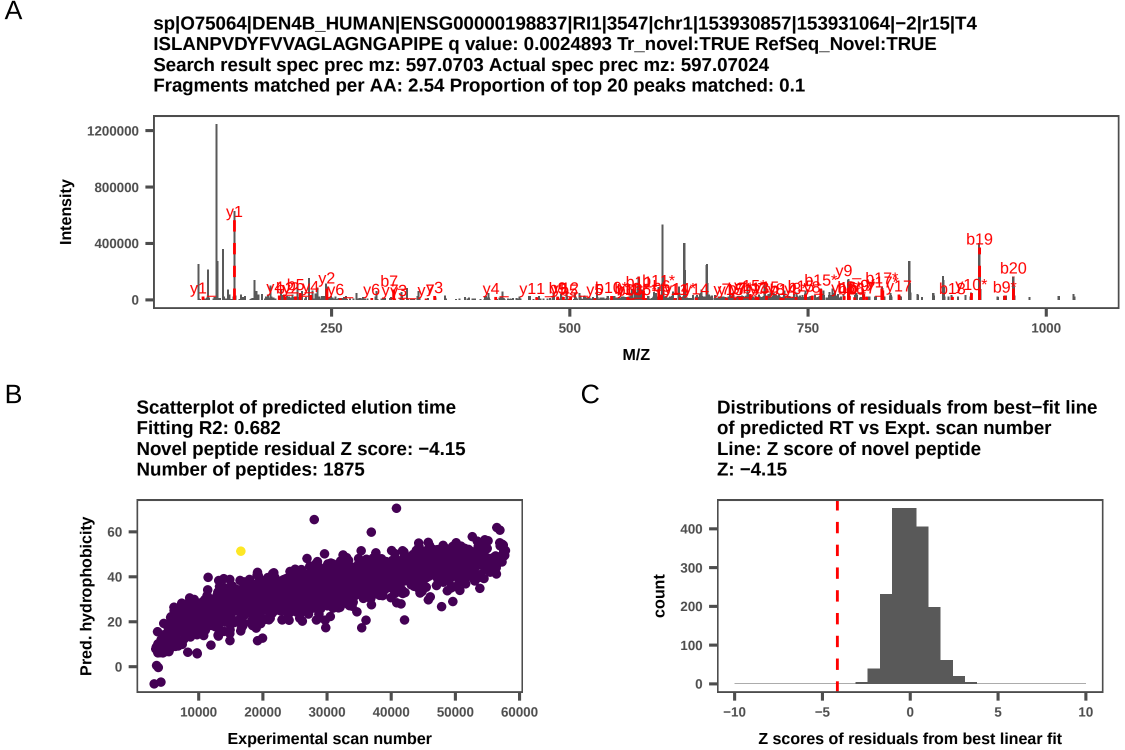
A
sp|O75064|DEN4B_HUMAN|ENSG00000198837|RI1|3547|chr1|153930857|153931064|−2|r15|T4
ISLANPVDYFVVAGLAGNGAPIPE q value: 0.0024893 Tr_novel:TRUE RefSeq_Novel:TRUE
Search result spec prec mz: 597.0703 Actual spec prec mz: 597.07024
Fragments matched per AA: 2.54 Proportion of top 20 peaks matched: 0.1
Intensity
1200000
800000
400000
0
y1
y2
y6
b7
y3
y4
y11
y9
b19
b20
b18
y10*
b9*
y17
b15*
b17*
250
500
750
1000
M/Z
B
Scatterplot of predicted elution time
Fitting R2: 0.682
Novel peptide residual Z score: −4.15
Number of peptides: 1875
Pred. hydrophobicity
60
40
20
0
10000
20000
30000
40000
50000
60000
Experimental scan number
C
Distributions of residuals from best−fit line
of predicted RT vs Expt. scan number
Line: Z score of novel peptide
Z: −4.15
count
400
300
200
100
0
−10
−5
0
5
10
Z scores of residuals from best linear fit
